# Model of negative affect induced by withdrawal from acute and chronic morphine administration in male mice

**DOI:** 10.1038/s41598-024-60759-3

**Published:** 2024-04-29

**Authors:** Dersu Ozdemir, Judith Meyer, Brigitte L. Kieffer, Emmanuel Darcq

**Affiliations:** 1 Université de Strasbourg (UNISTRA), INSERM UMR-S 1329, Strasbourg Translational Neuroscience and Psychiatry, Centre de Recherche en Biomédecine de Strasbourg, 1 rue Eugène Boeckel, 67084 Strasbourg Cedex, France; 2 Department of Psychiatry, Douglas Hospital Research Center, McGill University, Montreal, Canada

**Keywords:** Addiction, Social neuroscience

## Abstract

Opioid use disorder (OUD) is a chronic relapsing disorder that is a major burden for the lives of affected individuals, and society as a whole. Opioid withdrawal is characterized by strong physical symptoms, along with signs of negative affect. Negative affect due to opioid withdrawal is a major obstacle to recovery and relapse prevention. The mechanisms behind negative affect due to either spontaneous or antagonist-precipitated opioid withdrawal are not well known, and more animal models need be developed. Here, we present behavioral models of negative affect upon naloxone-precipitated morphine withdrawal in adult male mice. Social, anxiety, and despair-like deficits were investigated following naloxone administration in mice receiving morphine under three dosing regimens; acute, chronic constant dose and chronic escalating doses. Social behaviour in the three-chamber social preference test was decreased following withdrawal from chronic and escalating but not acute morphine. Anxiety-like behaviour in the open field was increased for all three treatments. Despair-like behaviour was increased following withdrawal from chronic and escalating but not acute morphine. Altogether, these animal models will contribute to study behavioural and neuronal circuitries involved in the several negative affective signs characterizing OUD.

## Introduction

Opioid use disorder (OUD) is a complex relapsing disorder following repeated opioid exposure, affecting 16 million people worldwide with an estimation of 120,000 deaths worldwide annually attributed to OUD^2^. OUD leads to significant impairment and poor life quality^1,2^. OUD is often described within a recurring three-stage framework comprised of binge/intoxication upon drug consumption, withdrawal and negative mood when access to the drug is limited, and preoccupation/anticipation with the drug until the next intoxication^3^. Withdrawal is particularly problematic in OUD, as avoidance of the negative emotional state by using the drugs is a major negative reinforcer for continued opioid use^4^. Opioid withdrawal syndrome is comprised of an intense acute phase that develops within minutes to several hours after a cessation or reduction in opioid use^5^. Acute opioid withdrawal is characterized by dysphoria and somatic signs such as nausea or vomiting; muscle aches; lacrimation, pupillary dilation, piloerection, or sweating, diarrhea, yawning, fever, or insomnia^5^. Acute withdrawal can also be induced by administration of an opioid antagonist such as naloxone and naltrexone after chronic opioid use. Naloxone is a life-saving drug used to reverse opioid overdose^6^. However, a concern with the administration of naloxone in an opioid-dependent state is the precipitation of the highly aversive emotional and somatic withdrawal symptoms. The severity of signs of withdrawal is correlated to continued opioid use and risk of relapse^7^. It is therefore critical to alleviate the acute withdrawal phase and its associated negative affective state in people with OUDs, and it remains key to maintain abstinence.

The neurobiological mechanisms underlying negative affect during naloxone-precipitated withdrawal are still not well understood, however animal models have proven an invaluable preclinical tool to help advance the field^8,9^. Many animal studies of addiction-like behaviours aim to model the rewarding aspects of OUD and/or on the negative reinforcement, ‘the dark side of addiction’^4^. Dysphoric mood during opioid withdrawal has been modelled in animals through avoidance of withdrawal-associated contexts, social deficits, and increased anxiety- and despair-like behaviours^8^. Most models of negative affect during opioid withdrawal are studied after chronic opioid exposure in order to induce the longer-term adaptations thought to be necessary for the development of a withdrawal signs^8^. However, few models address acute effects of morphine, though studies show that even a single administration of morphine can cause anxiety-like behaviours following naloxone precipitated withdrawal^10,11^. Additionally, animal models typically include escalating dose opioid administration to replicate the increased opioid intake pattern in humans following the development of tolerance, however it is not often shown that escalating dosing is necessary for the development of negative affect. It is therefore important to distinguish negative affective signs that develop as a result of repeated and escalating opioid administration, in an effort to better model patterns of opioid intake.

Here, we present models of negative affect during naloxone-precipitated withdrawal from acute, chronic, and escalating dose morphine. Following naloxone-precipitated withdrawal from three different patterns of morphine exposure, mice were placed in behavioural tests of negative affect including social, anxiety-like and despair on three separate days. We showed that acute, chronic and escalating morphine were sufficient to induce increased anxiety-like behaviour. However, only chronic morphine, at constant and escalating dosing, induced social and despair-like behavioural deficits, indicating these behaviours are chronic dosing dependent features of negative affect. The negative affective deficits did not require escalating dose of morphine, and chronic opioid exposure was sufficient for all signs of negative affect tested. Somatic withdrawal symptoms in response to naloxone increased in severity as morphine dosing increased across the three administration patterns. Remarkably, while the presence of somatic withdrawal signs is typically used as a control for the development of physical dependence to morphine, they were also found to be increased following an acute dose of morphine. Thus, this comparison highlights the importance of these animal models for examining the neural and behavioural circuits and to test new therapeutic strategies related to negative emotional processes in the context of OUD.

## Materials and methods

### Mice

Adult male C57Bl6/J mice (> 8 weeks old; Charles River) were housed in groups of 4–5 in a temperature and humidity-controlled room. Juvenile C57Bl/6J mice (< 7 weeks old; Charles River) were used as interactors for test mice in the social preference test. All experiments were performed in accordance with the ARRIVE guidelines. All procedures in this report were conducted in accordance with the relevant guidelines and regulation set forth by the Canadian Council of Animal Care and by the Animal Care Committees of McGill University/Douglas Mental Health University Institute and were also approved by the Regional Committee of Ethic in Animals Experiment of Strasbourg (CREMEAS).

### Drugs

**Figure 1:**
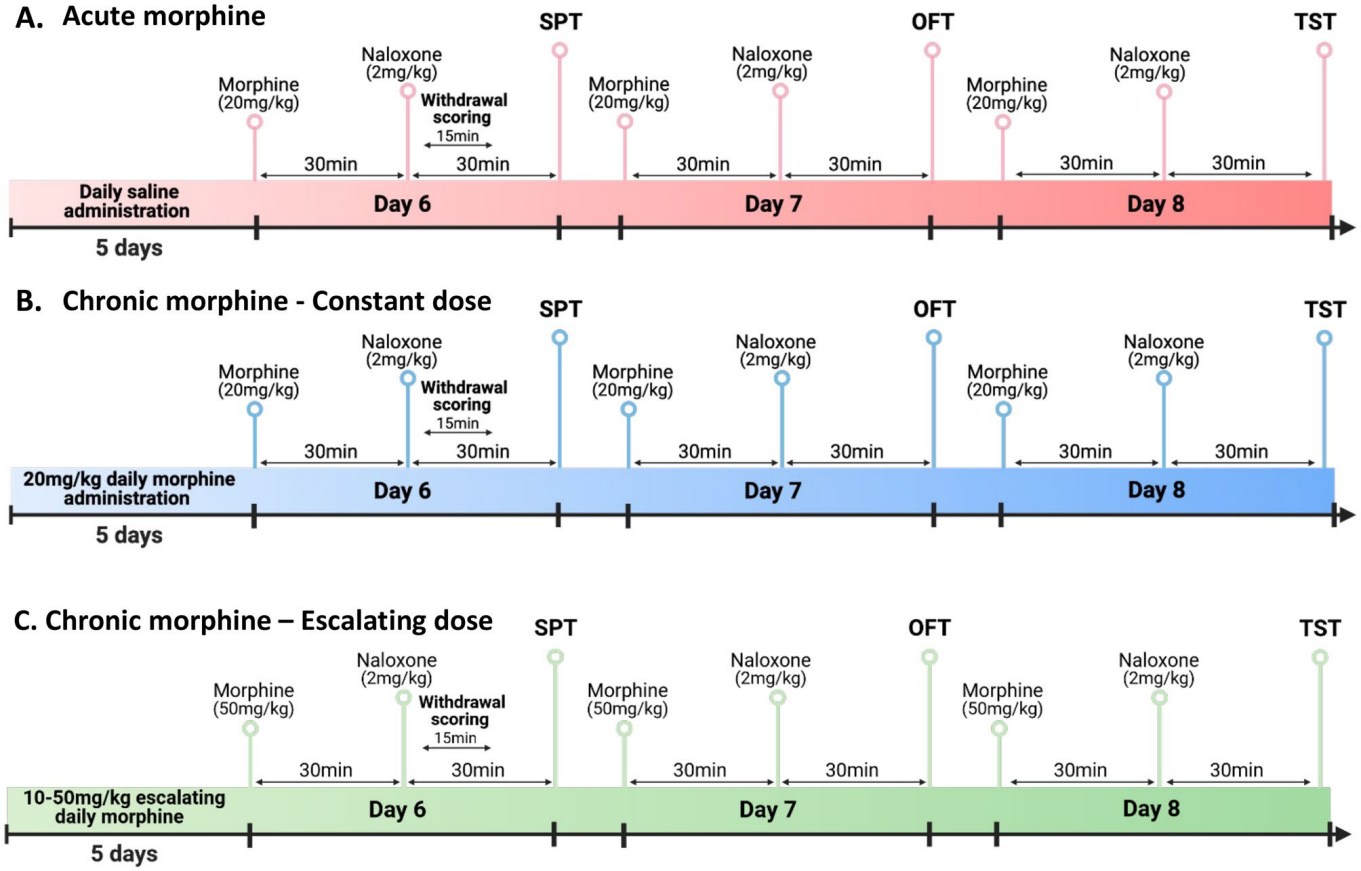
Experimental timeline of models of negative affect following naloxone-precipitated withdrawal from acute or chronic morphine. (**A**) Scheme of experimental timeline for naloxone-precipitated withdrawal from acute morphine. (**B**,**C**) Scheme of experimental timeline for naloxone-precipitated withdrawal from chronic morphine [with constant (**B**) or escalating (**C**) doses].

All the drugs were injected intraperitoneally (i.p.). In the acute morphine study (Fig. 1A), all mice received a saline injection (0.9%) once daily for 5 days. On day 6, mice received an acute injection of morphine (20 mg/kg) or saline (equivalent in volume), and 30 min later all mice received an injection of naloxone (2 mg/kg). In the chronic morphine study (Fig. 1B), mice received morphine (20 mg/kg) or equivalent in volume of saline once daily for 5 days. On day 6, mice received an injection of morphine (20 mg/kg) or saline, and 30 min later all mice received an injection of naloxone (2 mg/kg). In the escalating chronic morphine study (Fig. 1C), mice received morphine at escalating doses (day 1, 10 mg/kg; day 2, 20 mg/kg; day 3, 30 mg/kg; day 4, 40 mg/kg; day 5, 50 mg/kg) or equivalent in volume of saline once daily for 5 days. On day 6, mice received an injection of morphine (50 mg/kg) or saline, and 30 min later all mice received an injection of naloxone (2 mg/kg). In all experiments, drug administrations of day 6 were repeated on days 7 and 8.

### Behaviour of negative affect

In all studies, mice were placed in behavioural tests 30 min after naloxone injection on experimental days 6, 7 and 8. In all studies; on day 6, mice were placed in a social preference test (SPT)^12,13^, on day 7 an open field test (OFT)^14^, and on day 8 a tail suspension test (TST)^15^. Additionally, on day 6, mice were scored for somatic withdrawal signs for 15-min after naloxone. Detailed descriptions of behavioural procedures are in the Supplementary Information and as described^12–15^. This includes the description of precipitated withdrawal sign scoring, SPT, OFT, and TST.

### Statistics

Data are presented as mean ± SEM. Global withdrawal sign scoring and immobility time in the TST were represented across time and were analyzed using RM two-way ANOVAs. Significant main effects (*p* ≤ 0.05) were followed by Bonferroni’s multiple comparisons tests. All other experiments were first tested for normality with a Shapiro–Wilk test, followed by an unpaired t-test or Mann–Whitney U test. P-values < 0.05 were considered statistically significant. All data was analyzed using GraphPad Prism (v9) software. Please refer to Supplementary Information Table 1 for a description of all statistical tests.

## Results

### Three modes of morphine administration to study negative affect of withdrawal

Here, we designed models of negative affect during naloxone-precipitated withdrawal from acute (Fig. 1A), chronic, constant (Fig. 1B) and escalating (Fig. 1C) doses morphine. Following naloxone-precipitated withdrawal from three different patterns of morphine exposure, negative affect was evaluated using social, anxiety-like and despair behavioural tests on three separate days.

### Both acute and chronic morphine treatments induce signs of somatic withdrawal in naloxone administered mice

**Figure 2:**
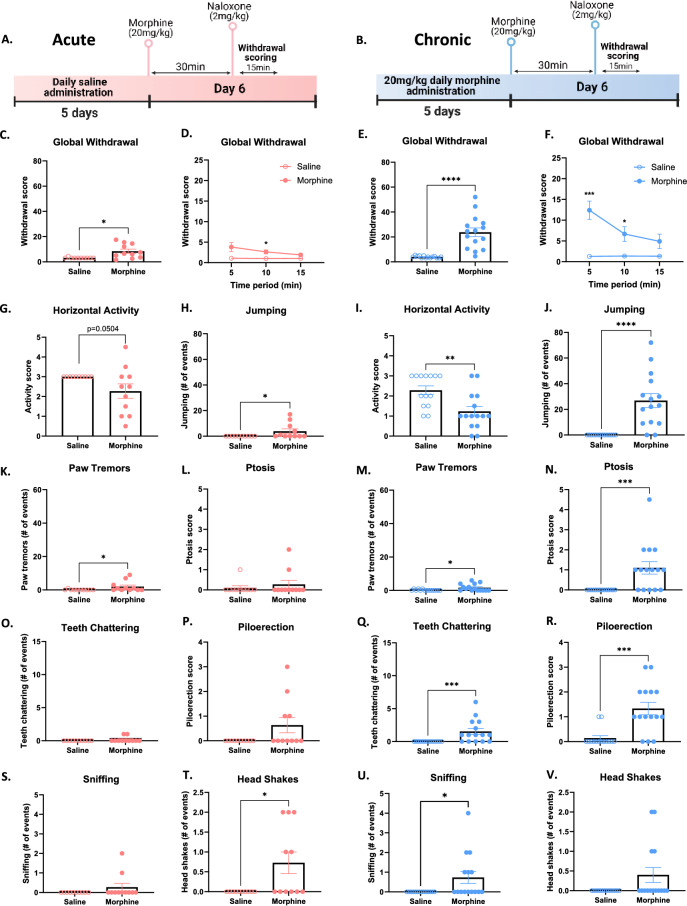
Naloxone-precipitated somatic withdrawal signs in acute and chronic morphine-administered mice (**A**,**B**) Schematic representation of experimental timelines of naloxone-precipitated withdrawal sign scoring for acute and chronic morphine-treated mice, respectively. (**C**,**E**) Increased global withdrawal sign score after naloxone injection in acute and chronic morphine treated mice, respectively. (**D**,**F**) Withdrawal sign scoring in 5-min time bins after naloxone injection in acute (**D**) and chronic (**F**) morphine treated mice. (**G**,**I**) Decreased horizontal activity score after naloxone injection in acute and chronic morphine treated mice, respectively. (**H**,**J**) Increased number of jumping events after naloxone injection in acute and chronic morphine treated mice, respectively. (**K**,**M**) Increased number of paw tremors after naloxone injection in acute and chronic morphine treated mice, respectively. (**L**,**N**) Ptosis score after naloxone injection is unaltered in acute (**L**) and increased in chronic (**N**) morphine treated mice. (**O**,**Q**) Number of teeth chattering events after naloxone injection is unaltered in acute (**O**) and increased in chronic (**Q**) morphine treated mice. (**P**,**R**) Piloerection score is not affected after naloxone injection in acute (**P**) and is increased in chronic morphine (**R**) treated mice. (**S**,**U**) Number of sniffing events is not affected after naloxone injection in acute (**S**) and is increased in chronic morphine (**U**) treated mice. (**T**,**V**) Number of head shakes is increased after naloxone injection in acute (**T**) but not in chronic morphine (**V**) treated mice. Data are represented as mean ± SEM. Dots represent individual values. *p < 0.05, **p < 0.01, ***p < 0.001, ****p < 0.0001. N = 10–15/group.

Somatic withdrawal signs are one of the most widely reported signs of naloxone-precipitated opioid withdrawal in humans, and have also been well described in rodent models^8^. In order to evaluate how different morphine regimens induce somatic withdrawal signs following naloxone injection, we carried out withdrawal sign scoring following acute (Fig. 1A) or 5-day chronic morphine dosing (Fig. 1B). Mice were injected with naloxone 30-min after morphine and immediately placed in a tall arena for 15-min while they were scored by observers blind to treatment group. Withdrawal from acute and chronic morphine induced a higher withdrawal sign score (see Supplementary Information methods for formula) compared to saline controls (Fig. 2C, *p = 0.01; Fig. 2E, t_27_ = 5.487, ****p < 0.0001). When withdrawal scores were analyzed over 5-min time bins, only chronic morphine mice displayed significantly increased withdrawal signs in the first 5-min, and both acute and chronic morphine treated mice differed significantly from saline controls from 5 to 10 min (Fig. 2D, main effect of treatment, F_(1, 19)_ = 8.63, **p < 0.01; Fig. 2F, main effect of treatment, F_(1, 27)_ = 31.13, ****p < 0.0001). At the end of the 15-min of withdrawal scoring, neither acute nor chronic morphine treated mice differed from their respective saline controls (Fig. 2D,F). Both acute and chronic precipitated morphine withdrawal induced typical signs of withdrawal including decreased horizontal activity (Fig. 2G, p = 0.0504; Fig. 2I, **p < 0.01), increased the number of jumping (Fig. 2H, *p < 0.05; Fig. 2J, ****p < 0.0001) and paw tremor events (Fig. 2K, *p < 0.05; Fig. 2M, *p < 0.05). Withdrawal from acute morphine induced no changes in ptosis, teeth chattering, piloerection or sniffing (Fig. 2L, p = 0.86; Fig. 2O, p = 0.48; Fig. 2P, p = 0.09; Fig. 2S, p = 0.48). Withdrawal from chronic morphine induced increased ptosis, teeth chattering, piloerection and sniffing events (Fig. 2N, ***p < 0.001; Fig. 2Q, ***p < 0.001; Fig. 2R, ***p < 0.001; Fig. 2U, *p < 0.05). Additionally, withdrawal from acute and not chronic increased the number of head shakes (Fig. 2T, *p < 0.05; Fig. 2, , p = 0.1). Finally, both acute and chronic morphine decreased rearing events and grooming events (Supplementary Information Fig. 1), the latter an indicator of decreased self-directed behaviour, often present in mouse models of negative affect such as the unpredictable chronic mild stress model^16^. These results show that both acute and chronic morphine induce increased signs of somatic withdrawal after naloxone.

### Chronic but not acute morphine treatment induces social deficits during naloxone precipitated withdrawal in mice

**Figure 3:**
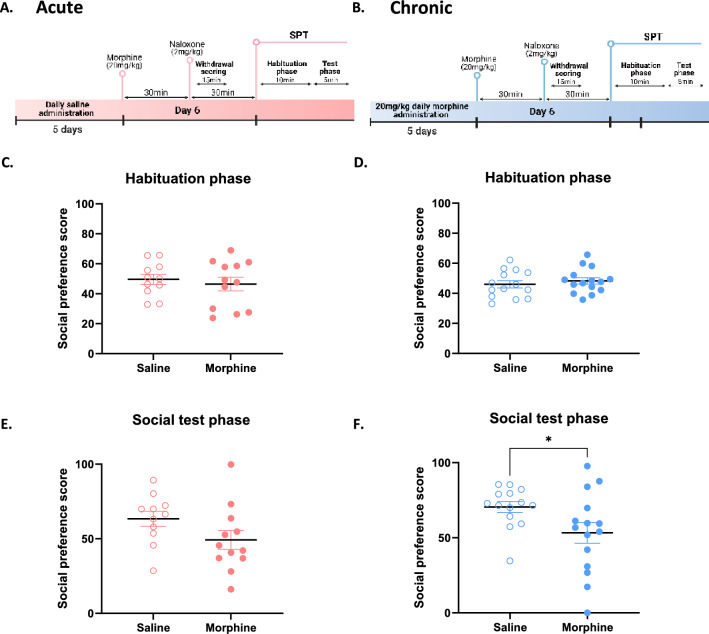
Naloxone-induced social behavioural deficits in chronic but not acute morphine-administered mice. (**A**,**B**) Schematic representation of experimental timelines of social preference test for acute (i.p.) and chronic (i.p.) morphine-treated mice, respectively. (**C**,**D**) No preference for the social-paired compartment in acute (**C**) or chronic (**D**) morphine treated mice 30 min after naloxone injection during the habituation phase of the social preference test. (**E**,**F**) Acute morphine induced (**E**) no difference in social preference compared to saline whereas chronic morphine (**F**) decreased preference for the social compartment when compared to saline 30 min after naloxone injection. Data are represented as mean ± SEM. Dots represent individual values. *p < 0.05. N = 10–15/group.

Social isolation during opioid withdrawal is a major impediment to recovery and relapse prevention^9^. We tested whether naloxone-precipitated withdrawal from acute or chronic morphine in mice could induce social deficits in a three-chamber social preference test (SPT). In this test, mice are given free access to 3 chambers throughout a 10-min habituation phase followed by a 5-min social test phase. During the habituation phase, empty downturned cups were placed in opposite chambers, while during the social phase an unfamiliar juvenile mouse was placed under one cup in the previously assigned social compartment. A social preference score is then calculated taking into account the time passed in the social vs the object compartment. Mice treated prior with acute or chronic morphine were placed in the apparatus with empty cups 30-min following naloxone administration for the habituation phase of the SPT (Fig. 3A,B). Acute or chronic morphine treated mice showed no preference for either chamber during the habituation phase (Fig. 3C, t_21_ = 0.55, p = 0.59; Fig. 3D, t_27_ = 0.71, p = 0.48). During the test phase, mice withdrawn from acute morphine showed no difference in social preference from saline controls (Fig. 3E; t_21_ = 1.72, p = 0.1;). Mice withdrawn from chronic morphine showed a lower social preference score compared to saline controls, revealing social behavioural deficits (Fig. 3F, t_27_ = 2.16, *p < 0.05). These results suggest that precipitated-withdrawal from chronic but not acute morphine is sufficient to induce social deficits.

### Naloxone-precipitated withdrawal from both acute and chronic morphine increases anxiety-like behaviour in the open field test

**Figure 4:**
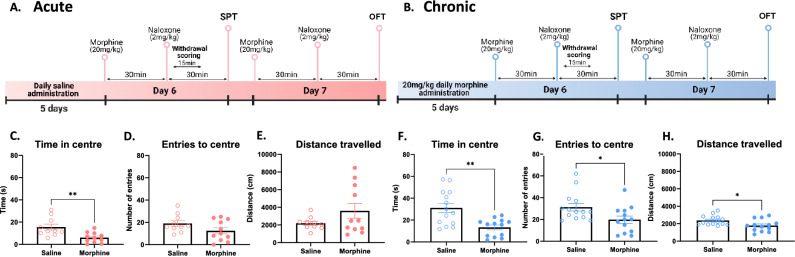
Naloxone-induced anxiety-like behaviour in acute and chronic morphine-administered mice. (**A**,**B**) Schematic representation of experimental timelines of open field test for acute (i.p.) and chronic (i.p.) morphine-treated mice, respectively. (**C**,**F**) Decreased time in centre zone in the open field test during naloxone-precipitated withdrawal from acute and chronic morphine, respectively. (**D**,**E**) No effect of precipitated withdrawal from acute morphine on entries to centre and distance travelled in the open field test, respectively. (**G**,**H**) Decreased entries to centre zone and distance travelled in the open field test during naloxone-precipitated withdrawal from chronic morphine. Data are represented as mean ± SEM. Dots represent individual values. *p < 0.05, **p < 0.01. N = 10–15/group.

In humans, anxiety is a commonly reported sign of negative affect during naloxone-precipitated opioid withdrawal^8^. We therefore sought to study how anxiety-like behaviour in the open field test (OFT) is affected during naloxone-precipitated withdrawal in our models of acute and chronic morphine withdrawal. To do this, mice treated prior with acute or chronic morphine were placed in an open field arena 30-min following naloxone administration (Fig. 4A,B). For 15 min, the time spent in the center of the arena, the number of entries to the center zone and the total distance travelled were recorded. Acute morphine treated mice spent less time in the center compared to saline controls during naloxone-precipitated withdrawal (Fig. 4C; t_19_ = 3.25, **p < 0.01), demonstrating increased anxiety-like behaviour with no effect on locomotion (Fig. 4E; p = 0.70). There was no effect of withdrawal from acute morphine on the number of entries to the center of the arena (Fig. 4D; t_19_ = 1.73, p = 0.0990). Chronic morphine treated mice also display increased anxiety-like behaviour during naloxone-precipitated withdrawal, demonstrated by decreased time in the center (Fig. 4F; t_25_ = 3.65, **p < 0.01) and number entries to the center (Fig. 4G; *p < 0.05). However, precipitated withdrawal from chronic morphine also decreased locomotor activity demonstrated by lower distance travelled (Fig. 4H; t_(25)_ = 2.48, *p < 0.05). Our results suggest that precipitated-withdrawal from acute and chronic morphine increase anxiety-like behaviour in the OFT, modeling a commonly reported symptom present during naloxone-precipitated withdrawal in humans.

### Chronic but not acute morphine treatment induces despair-like behaviour during naloxone precipitated withdrawal in mice

**Figure 5:**
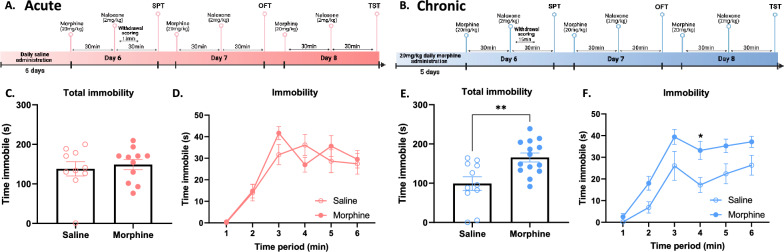
Naloxone-induced despair-like behaviour in chronic but not acute morphine-administered mice (**A**,**B**) Schematic representation of experimental timelines of tail suspension test for acute (i.p.) and chronic (i.p.) morphine-treated mice, respectively. (**C**,**D**) No effect naloxone-precipitated withdrawal from acute morphine on immobility time in the tail suspension test. (**C**,**D**) Increased immobility time in the tail suspension test during naloxone-precipitated withdrawal from chronic morphine. Data are represented as mean ± SEM. Dots represent individual values. *p < 0.05, **p < 0.01. N = 10–15/group.

To further probe the effect of morphine withdrawal on the spectrum of negative affect, we investigated the effect of naloxone-precipitated withdrawal from acute and chronic morphine on despair-like behaviour measured by the TST. Thirty minutes after a naloxone injection to precipitate withdrawal from acute or chronic morphine, mice were suspended upside-down from a hook by a paper clip attached to the tip of their tails (Fig. 5A,B). Total immobility time over 6 min was measured automatically by the apparatus. Mice withdrawn from acute morphine showed no difference in immobility time from saline controls (Fig. 5C, t_19_ = 0.50, p = 0.62; Fig. 5D, main effect of treatment, F_(1, 19)_ = 0.25, p = 0.62). However, we found that precipitated withdrawal from chronic morphine increased immobility time, showing deficits in active coping in the tail suspension test (Fig. 5E, t_23_ = 3.32, **p < 0.01; Fig. 5F, main effect of treatment, F_(1, 23)_ = 11.01, **p < 0.01). These results suggest that precipitated-withdrawal from chronic but not acute morphine is necessary to induce despair-like behaviour measured by the TST.

### Naloxone-precipitated withdrawal from escalating chronic morphine induces somatic withdrawal signs

**Figure 6:**
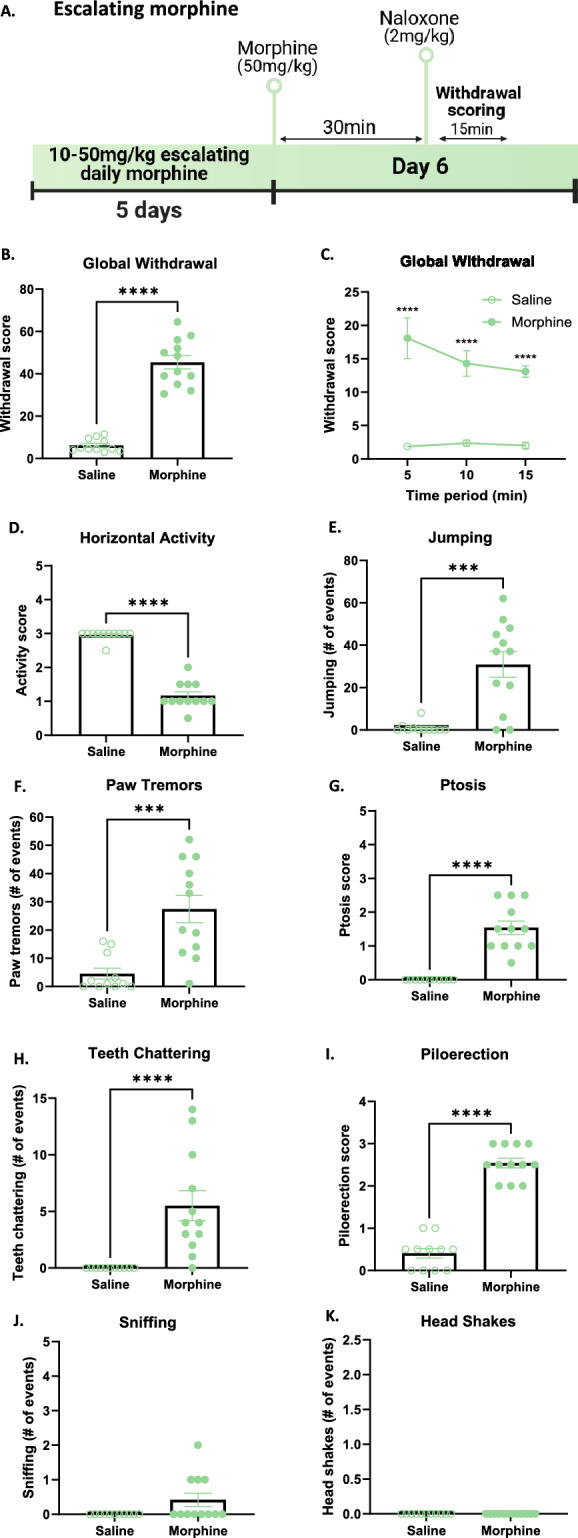
Naloxone-precipitated somatic withdrawal signs in chronic escalating morphine-administered mice (**A**) Schematic representation of experimental timeline of naloxone-precipitated withdrawal sign scoring for chronic escalating morphine-treated mice. (**B**) Increased global withdrawal sign score after naloxone injection in escalating morphine treated mice. (**C**) Withdrawal sign scoring in 5-min time bins after naloxone injection in escalating morphine treated mice. (**D**) Decreased horizontal activity score after naloxone injection in escalating morphine treated mice. Increased number of jumping (**E**), paw tremors (**F**), teeth chattering events (**H**) after naloxone injection in escalating morphine treated mice. Ptosis (**G**) and piloerection (**I**) scores are increased after naloxone injection in escalating morphine treated mice. Number of sniffing (**J**) and head shake (**K**) events is not affected after naloxone injection in chronic escalating morphine treated mice. Data are represented as mean ± SEM. Dots represent individual values. ***p < 0.001, ****p < 0.0001. N = 11–13/group.

One of the major characteristics of opioid addiction is a development of tolerance over time, leading to an escalation of opioid dose used^4^. To model this pattern of opioid intake, we used a mouse model of an escalating 5-day chronic treatment of morphine in order to evaluate the severity of somatic withdrawal signs following naloxone injection (Fig. 6A). Mice were injected with naloxone 30-min after morphine and somatic withdrawal signs were scored. Withdrawal from chronic escalating morphine induced a high global withdrawal sign score compared to saline controls (Fig. 6B, U = 0, ****p < 0.0001). When withdrawal scores were analyzed over 5-min time bins, chronic escalating morphine treated mice displayed significantly increased withdrawal signs compared to saline controls over all 5-min time bins (Fig. 6C, main effect of treatment, F_(1, 21)_ = 131.1, ****p < 0.0001). At the end of the 30 min of withdrawal scoring, escalating morphine treated mice did not differ from saline controls (Supplementary Information Fig. 2). Precipitated withdrawal from chronic escalating morphine induced typical signs of withdrawal including decreased horizontal activity (Fig. 6D, U = 0, ****p < 0.0001), increased the number of jumping (Fig. 6E, U = 15, ***p < 0.001), paw tremor (Fig. 6F, U = 13.5, ***p < 0.001) and teeth chattering events (Fig. 6H, U = 5.5, ****p < 0.0001). Withdrawal from chronic escalating morphine induced increased ptosis and piloerection scores (Fig. 6G, U = 0, ****p < 0.0001; Fig. 6I, U = 0, ****p < 0.0001). Additionally, withdrawal from chronic escalating morphine had no significant effect on the number of sniffing and head shake events (Fig. 6J, U = 44, p = 0.09; Fig. 6K, U = 66, p > 0.99). Finally, escalating morphine decreased rearing and grooming events (Supplementary Information Fig. 1). These results show that as with acute and chronic morphine, escalating morphine induces increased signs of somatic withdrawal after naloxone. Escalating morphine treated mice displayed higher and more long-lasting global withdrawal signs when compared to acute and chronic constant morphine treated mice. Additionally, escalating treatment increased jumping, paw tremor, teeth chattering and ptosis scores when compared to acute and constant morphine. While acute morphine increased head shakes after naloxone, and constant morphine increased sniffing, escalating morphine had no significant effect on these signs, demonstrating a dosing paradigm-dependent apparition of withdrawal signs.

### Naloxone-precipitated withdrawal from escalating chronic morphine induces social deficits, anxiety-like and despair-like behaviour

**Figure 7:**
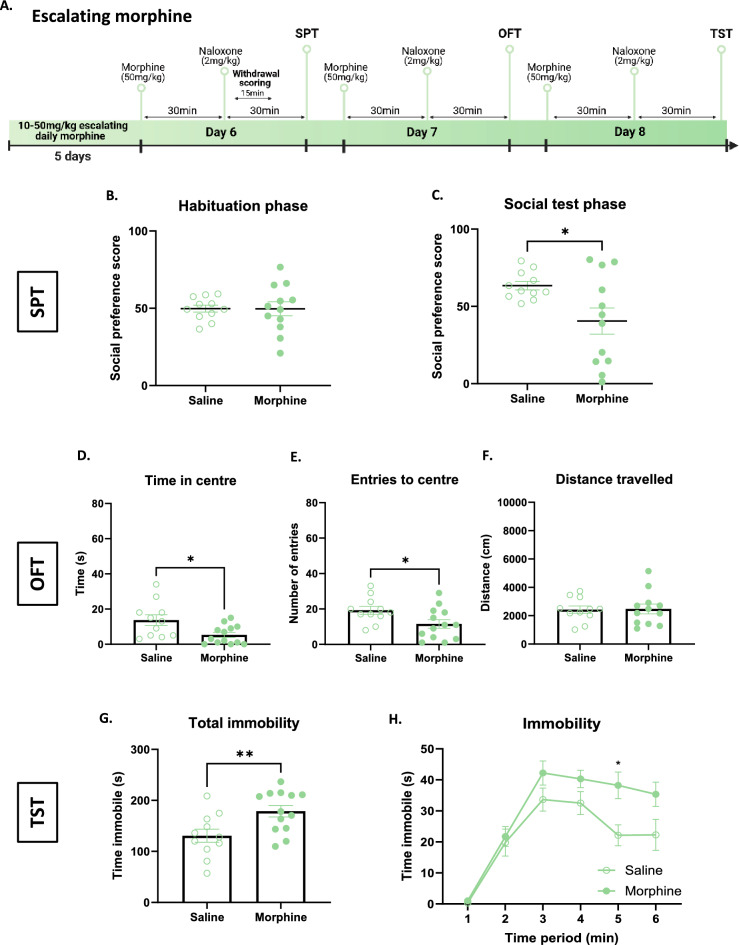
Negative affect during naloxone-precipitated withdrawal in mice receiving a chronic escalating dose morphine regimen. (**A**) Scheme of experimental procedure. (**B**,**C**) Decreased social preference during naloxone-precipitated withdrawal from escalating morphine. (**D**–**F**) Decreased time and entries to the centre zone in the open field following naloxone-precipitated withdrawal from escalating morphine with no effect on distance travelled. (**H**,**I**) Increased immobility time in the tail suspension test during naloxone-precipitated withdrawal from chronic escalating morphine. *p < 0.05, **p < 0.01. Data are represented as mean ± SEM. Dots represent individual values. N = 11–13/group.

Next, we determine whether the negative affect deficits are also present after an escalating opioid treatment. To do so we used a mouse model of an escalating 5-day chronic treatment of morphine and tested negative affect during naloxone precipitated withdrawal (Fig. 7A). After a 5-day escalating morphine treatment, mice were injected with naloxone and subjected to a series of behavioural tests probing negative affect over the course of 3 days. Social behaviour was tested on day 6 with an SPT 30 min after naloxone injection. Both saline and morphine-treated mice showed no preference for either chamber during the habituation phase (Fig. 7B; t_21_ = 0.03, p = 0.98). Precipitated withdrawal from escalating morphine decreased time spent in the social chamber when compared to saline controls (Fig. 7C; t_21_ = 2.48, *p < 0.05). On day 7, mice were then placed in an OFT 30-min after naloxone to test anxiety-like behaviour. Escalating chronic morphine treated mice displayed increased anxiety-like behaviour during naloxone-precipitated withdrawal, demonstrated by decreased time in the center (Fig. 7D; t_22_ = 2.63, *p < 0.05) and number entries to the center of the arena (Fig. 7E; t_22_ = 2.30, *p < 0.05;), with no effect on locomotion (Fig. 7F; t_21_ = 0.11, p = 0.92). Finally, on day 8 mice were placed in a TST to measure despair-like behaviour 30-min after naloxone. Precipitated withdrawal from escalating chronic morphine increased immobility time, demonstrating deficits in active coping in the tail suspension test (Fig. 7G, t_22_ = 2.83, **p < 0.01; Fig. 7H, main effect of treatment, F_1, 22_ = 7.99, **p < 0.01). These results suggest that this model of naloxone precipitated withdrawal from chronic escalating morphine, as after chronic constant morphine, is able to model social deficits and anxiety-like and despair-like behavioural signs of negative affect.

## Discussion

Here, we established and described a model of adverse effects during and after naloxone-induced withdrawal from morphine with acute, chronic, and increasing regiments. Mice were subjected to behavioural measures of negative affect, which included social interaction, anxiety-like, and despairing tasks. We demonstrated that enhanced anxiety-like behaviour could be induced by both acute and chronic morphine use. Nevertheless, social and depressive-like behavioural abnormalities were only brought on by chronic dosing, suggesting that these behaviours are chronic dosing dependent aspects of negative affect. Chronic opioid exposure was sufficient to produce all tested indicators of negative affect, and increasing the amount of morphine did not increase the negative affective impairments.

### Other animal models of antagonist-precipitated withdrawal

When an opioid-dependent individual receives an opioid receptor antagonist, it causes a withdrawal episode^17^. Naloxone and naltrexone are the two primary opioid antagonists that are commonly utilized in the clinical context. One of the three FDA-approved drugs for treating OUDs is naltrexone, which is also used to reduce opioid overdose deaths^18^. Exposure to antagonists causes and intensifies the withdrawal symptoms that naturally happen when the opioid medicine wears off. Withdrawal symptoms (somatic and negative affect) can be enhanced and temporally controlled using antagonist precipitated opioid withdrawal models. Inducing somatic withdrawal symptoms in dogs and rodents through antagonist-precipitated withdrawal has been a popular method for confirming the existence of physical dependency^19–23^. After just one opioid encounter, agonist-precipitated withdrawal symptoms can be quantified^24^. This phenomena was found in dogs exhibiting antagonist-precipitated somatic withdrawal-like symptoms one hour following a single morphine administration^19^ and in human and rodents^24–26^. The amount of withdrawal symptoms following an acute dosage is largely influenced by the agonist and antagonist doses as well as the time between the two administrations^11,24^. When an antagonist is administered following an acute morphine dose, anxiety-like reactions are enhanced for up to 80 days following the acute withdrawal phase (two hours)^10,11^. And as here, several symptoms of antagonist-precipitated withdrawal, such as aversion to withdrawal-conditioned taste and location avoidance^10,27–29^, increased anxiety-like responses^10,11,27,30–34^, and anhedonia and reward deficits^35–37^, that were observed under chronic opioid treatments were also present following a single, unusual acute dosage. Altogether, these and our studies suggest that negative affect after withdrawal from an acute opioid dose may be similar to signs of withdrawal from chronic opioid.

### Somatic precipitated withdrawal signs are present after both acute and chronic morphine

Typically, the presence of somatic withdrawal signs has been used in animal models of addiction to verify the presence of physical dependence^38^. Our study demonstrates that one acute dose of morphine 30 min prior to naloxone is sufficient to induce somatic withdrawal signs, therefore the use of naloxone as an indicator of dependence that develops as a result of chronic exposure to morphine might not be always necessary. However, chronic morphine withdrawal induced higher severity of signs of withdrawal, likely reflecting the adaptations that occur at the cellular level over a chronic treatment. Finally, chronic escalating morphine withdrawal induced the most severe and long-lasting somatic withdrawal signs, demonstrating a dose-dependent increase in severity and duration of withdrawal signs. Importantly, neither acute nor chronic constant morphine treated mice differed from their saline controls by the end of the 15-min scoring period, while chronic escalating morphine treated mice did not differ from controls at the 25-min time point (Supplementary Information Fig. 2). This demonstrates that somatic withdrawal signs were absent 30 min after naloxone, preventing interference with behavioural tests of negative affect.

### Social and despair-deficits

Social deficits are a hampering symptom of opioid withdrawal. The literature focused on social behavioural deficits following protracted abstinence in mice^38,39^, with few studies on social behaviour following short-term opioid withdrawal^12^. In one study, thirty minutes after naloxone injection, time in the social zone during the social preference test was reduced by naloxone-precipitated morphine withdrawal^12^. While our results are coherent with this previous study, social interaction deficits may be more easily and robustly detected using live mouse tracker technology allowing a detection of social interaction on an automatic and unbiased manner^40^. Importantly, only precipitated withdrawal from chronic morphine could induce social and despair-like deficits. These signs are appropriate to test for adaptations after long-term opioid use and mechanisms behind withdrawal.

### Anxiety-like behaviour deficits

Antagonist-precipitated opioid withdrawal from chronic opioids induces anxiety-like behavioural deficits. The unpleasant withdrawal experience was often associated to a cue or context^23,41,42^. Anxiety-like deficits were also found after antagonist-precipitated withdrawal in the elevated plus maze^34,43^. Furthermore, this anxiety-like behavioural deficits are long lasting, as Rothwell and colleagues showed that naloxone-precipitated withdrawal increased anxiety-like responses until 80 days after the exposure to one morphine (10 mg/kg) injection^10^. Anxiety-like behaviour is a commonly detected sign of negative affect in many rodent models of withdrawal^8^. Here, anxiety is detected following withdrawal from each morphine treatment, demonstrating a sensitive sign of negative affect present after withdrawal from even acute morphine. In the literature, anxiety is present after short-term withdrawal and protracted abstinence from acute and chronic opioid exposure^8^. Withdrawal from an acute morphine dose as presented here could serve as an appropriate model to detect early adaptations to opioids that are revealed by mu-opioid receptor antagonism.

### Improvement and use of the model

To more accurately mimic patterns of opioid intake, it is crucial to differentiate between unpleasant emotional signals that arise from recurrent and increasing opioid administration. It may be important to develop a model of antagonist precipitated withdrawal after voluntary intake of opioid to better mimic the pattern of opioid consumption in humans. Other studies, show that there was no discernible impact on heroin-seeking behaviour in dependent or non-dependent rats when opioid antagonists (naltrexone or naloxone) created an unpleasant condition^44–46^. Naloxone-precipitated somatic signs of withdrawal were detected in rats that self-administered heroin^47^. Naloxone also increases voluntary heroin intake in operant self-administration procedure^48,49^. It is also important to note that the number of naloxone administrations may impact the results, and thus the intensity of negative affect deficits may depend of the order of the behavioral tests. Another limitation of the study is that sex differences were not studied as only male mice were used. Indeed, sex may influence negative affective deficits. It was shown previously, that sex influences some signs of despair or anxiety after protracted withdrawal^50^. Another study shows no main difference in withdrawal signs between males and females after naloxone-precipitated withdrawal from acute morphine (besides number of fecal boli, paw tremor and weight loss)^7^. Thus, further studies will be necessary to determine the impact of the sex on negative affect after opioid withdrawal.

Altogether, the model describes in our study will be important to dissect the neural and behavioural circuits and to test new therapeutic strategy involved in the development of negative affect in the context of OUD.

### Supplementary Information

Supplementary Information.

## Data Availability

The datasets used and analyzed during the current study are available from the corresponding author on reasonable request.
