# Supplementary material for: Model of negative affect induced by withdrawal from acute and chronic morphine administration in male mice
Source: Sci Rep. 2024 Apr 29;14:9767. doi: 10.1038/s41598-024-60759-3 (PMC11059349; doi:10.1038/s41598-024-60759-3)
Supplement: Supplementary file 1 — Supplementary Information. [file 41598_2024_60759_MOESM1_ESM.docx]

***Supplementary Information***

**Mouse model of negative affect induced by withdrawal from acute and chronic morphine administration in male mice**

**Author Affiliations**

Dersu Ozdemir^1^, Judith Meyer^1^, Brigitte L. Kieffer ^1,2^, Emmanuel Darcq ^1,2^

^1^ Université de Strasbourg (UNISTRA), INSERM UMR-S 1329, Strasbourg Translational Neuroscience and Psychiatry, Centre de Recherche en Biomédecine de Strasbourg, France.

^2^ Douglas Hospital Research Center, Department of Psychiatry, McGill University, Montreal, Canada.

**Corresponding author:**

Emmanuel Darcq, PhD

Centre de Recherche en Biomédecine de Strasbourg

1 rue Eugène Boeckel

67084 Strasbourg Cedex France

Tel +33 3 68 85 36 53

[edarcq@unistra.fr](mailto:edarcq@unistra.fr)

**Supplementary methods**

***Housing.*** Mice were housed 4-5 per cage under a 12-h light/dark cycle (lights on at 7:00am) in a temperature- and humidity-controlled room. Test mice were 2-3 months old at the during testing, and interactor mice for the social preference test were 6-7 weeks old during testing. Food and water were available ad libitum. Mice were allowed to habituate to the housing for 1-week after delivery at the housing facility. For each behavioural test, mice were brought in the testing room 45 minutes before for habituation. To minimize stress induced by first experimental manipulation, mice were handled for 3 consecutive days before the start of an experiment.

***Drugs.*** All drugs in this study were administered at a volume of 0.1 ml/10g via intraperitoneal (i.p.) injection. As a control for morphine, 0.9% saline solution (sterile NaCl, B. Braun) was administered. Morphine sulfate pentahydrate (provided by NIH NIDA Drug Supply Program, catalog # 9300-001) was dissolved in 0.9% saline solution. Naloxone hydrochloride dihydrate (N7758, Sigma-Aldrich) was dissolved in 0.9% saline solution and used the day of preparation.

***Naloxone-precipitated withdrawal sign scoring.*** Mice were injected with naloxone 30 minutes after morphine and individually placed in clear Plexiglass chambers (30cm x 14cm x 30cm). Experimenters blind to the treatment group scored physical withdrawal signs as described previously^1,2^. Somatic signs of withdrawal were scored in bins of 5 minutes across a 15-minute session. We scored the numbers of wet dog head shakes, paw tremors, teeth chattering, jumps, body tremors, and sniffing episodes. Ptosis and piloerection were scored 1 for appearance, 0 for non-appearance, and 0.5 for partial appearance within 5 min bins. Locomotor activity over 5 min periods was rated 0 for inactivity, 1 for moderate activity and 2 for high activity (with the possibility of intermediate values 0.5 and 1.5), with the final score for locomotor activity a sum of the score over 5-min periods. A global withdrawal score was calculated for each animal giving each individual sign a relative weight: 0.5 for each episode of jump, paw tremor, body tremor, sniffing and head shake; and 1 for the presence of ptosis, piloerection, and teeth chattering per 5 min observation periods as follows: global score = 6-activity + (0.5 x number of jumps) + (0.5 x number of sniffings) + (0.5 x number of paw tremors) + (0.5 x number of body tremors) + (0.5 x number of head shakes) + (1 x ptosis) + (1 x teeth chattering) + (1 x piloerection). The number of grooming and rearing events were also scored but not included in the global withdrawal sign calculation.

***Social Preference Test (SPT).*** We adapted the protocol for the SPT from recent studies^3,4^. The SPT apparatus comprised of a 3-compartment box made in-house (58cm x 22cm; 22cm for each social-paired and object-paired compartment separated by a 9.5cm x 22cm central compartment; A closing mechanism allowed to separate each compartment. Juvenile interactor mice were habituated daily for three days prior to the test to being placed under the cups in the apparatus for 10 minutes. On the test day, test mice were exposed to the apparatus (indirectly lit at 25 Lux) for 10 minutes in the empty arena for the habituation phase. At the end of the habituation phase, mice were enclosed in the central compartment and two cups (diameter of bottom of cup 10cm, top diameter 7cm) were placed in each compartment. The juvenile interactor was then placed under the cup in the social-paired compartment. Mice were then given free access to the chambers during the 5-min social test phase, in which an empty cup remained in the object-paired compartment. The social-paired compartment was counterbalanced between conditions. Each interactor was used for no more than 2 test sessions. Times spent in the social zone and object zone were recorded during habituation and social test via AnyMaze software. The ratio of time spent in the social compartment vs the object compartment was used to determine a social preference score for the habituation phase and for the social test phase (time in social compartment/(time in social compartment + object compartment))^3^. An increased social preference score was used as an index of sociability ^4^.

***Open Field Test (OFT).*** For the OFT as described ^5^, an automated open field apparatus (SuperFlex Open Field, Omnitech Electronics, Inc) was used. Each arena (41x41cm) was sealed from the rest of the room to leave each mouse undisturbed from the exterior environment during the test. Mice were placed in a corner of the arena and distance travelled, and time and entries to the center zone (10x10cm) were recorded. For all experiments, the luminosity was set at 220 Lux.

***Tail Suspension Test (TST).*** For the TST, an automated tail-suspension apparatus (Bioseb, USA) was used to measure immobility. Mice were suspended by the tail, using surgical adhesive tape and a paper clip, to a hook connected to a strain gauge that transmitted the animal's movements to a central unit. The total duration of immobility was automatically measured during the 6-min test. Mice that climbed up their tail during the test were excluded from analysis^6^.

**Supplementary figures**

**D.**

**C.**

**A.**

**B.**

**F.**

**E.**

**Supplementary figure 1: Decreased stereotypical behaviours following naloxone precipitated withdrawal from acute, chronic and escalating morphine administration. (A, C, E)** Decreased number of rearing events during naloxone-precipitated withdrawal sign scoring after acute, chronic and escalating chronic morphine, respectively. **(B, D, F)** Decreased number of self-grooming events during naloxone-precipitated withdrawal sign scoring after acute, chronic and escalating morphine, respectively. *p<0.05, **p<0.01, ****p<0.0001. N = 10-15/group.

**Supplementary figure 2: Naloxone-precipitated somatic withdrawal scoring over 30 minutes in chronic escalating morphine-administered mice.** Withdrawal sign scoring in 5-minute time bins for 30 minutes after naloxone injection in chronic escalating morphine treated mice shows somatic withdrawal signs are no longer observed in the 25 and 30–minute time bins. *p<0.05, ***p<0.001, ****p<0.0001. N = 11-13/group.

# Supplementary Table

# Supplementary table 1: Detailed statistics of all figures.

# References

1 Le Merrer, J. *et al.* Protracted abstinence from distinct drugs of abuse shows regulation of a common gene network. *Addict Biol* **17**, 1-12, doi:10.1111/j.1369-1600.2011.00365.x (2012).

2 Boulos, L. J. *et al.* Mu opioid receptors in the medial habenula contribute to naloxone aversion. *Neuropsychopharmacology* **45**, 247-255, doi:10.1038/s41386-019-0395-7 (2020).

3 Valentinova, K. *et al.* Morphine withdrawal recruits lateral habenula cytokine signaling to reduce synaptic excitation and sociability. *Nat Neurosci* **22**, 1053-1056, doi:10.1038/s41593-019-0421-4 (2019).

4 Allain, F., Carter, M., Dumas, S., Darcq, E. & Kieffer, B. L. The mu opioid receptor and the orphan receptor GPR151 contribute to social reward in the habenula. *Sci Rep* **12**, 20234, doi:10.1038/s41598-022-24395-z (2022).

5 Bailly, J. *et al.* Habenular Neurons Expressing Mu Opioid Receptors Promote Negative Affect in a Projection-Specific Manner. *Biol Psychiatry* **93**, 1108-1117, doi:10.1016/j.biopsych.2022.09.013 (2023).

6 Welsch, L. *et al.* Mu Opioid Receptor-Expressing Neurons in the Dorsal Raphe Nucleus Are Involved in Reward Processing and Affective Behaviors. *Biol Psychiatry*, doi:10.1016/j.biopsych.2023.05.019 (2023).
